# Chitosan Based Biodegradable Composite for Antibacterial Food Packaging Application

**DOI:** 10.3390/polym15102235

**Published:** 2023-05-09

**Authors:** Andre Jiang, Rajkumar Patel, Bandana Padhan, Supriya Palimkar, Padmaja Galgali, Arindam Adhikari, Imre Varga, Madhumita Patel

**Affiliations:** 1 Department of Chemical Engineering, The Cooper Union for the Advancement of Science and Art, New York, NY 10003, USA; 2 Energy & Environmental Science and Engineering (EESE), Integrated Science and Engineering Division (ISED), Underwood International College, Yonsei University, 85 Songdogwahak-ro, Yeonsugu, Incheon 21938, Republic of Korea; rajkumar@yonsei.ac.kr; 3 Department of Biotechnology, School of Life Science and Biotechnology, Adamas University, Kolkata 700126, West Bengal, India; 4 Aadarsh Innovations, Balewadi, Pune 411045, Maharashtra, India; 5 Institute of Chemistry, Eötvös Loránd University, 1117 Budapest, Hungary; 6 Department of Chemistry and Nanoscience, Ewha Womans University, 52 Ewhayeodae-gil, Seodaemun-gu, Seoul 03760, Republic of Korea

**Keywords:** chitosan, composites, food packaging, biodegradable chitosan film, antimicrobial activity

## Abstract

A recent focus on the development of biobased polymer packaging films has come about in response to the environmental hazards caused by petroleum-based, nonbiodegradable packaging materials. Among biopolymers, chitosan is one of the most popular due to its biocompatibility, biodegradability, antibacterial properties, and ease of use. Due to its ability to inhibit gram-negative and gram-positive bacteria, yeast, and foodborne filamentous fungi, chitosan is a suitable biopolymer for developing food packaging. However, more than the chitosan is required for active packaging. In this review, we summarize chitosan composites which show active packaging and improves food storage condition and extends its shelf life. Active compounds such as essential oils and phenolic compounds with chitosan are reviewed. Moreover, composites with polysaccharides and various nanoparticles are also summarized. This review provides valuable information for selecting a composite that enhances shelf life and other functional qualities when embedding chitosan. Furthermore, this report will provide directions for the development of novel biodegradable food packaging materials.

## 1. Introduction

Products made from synthetic plastics, such as disposable grocery bags, are used daily. However, negative consequences of the nonrenewable, petroleum-based synthetic plastics have become increasingly known [1,2]. Synthetic plastics are nonbiodegradable and can take thousands of years to decompose. Although these synthetic polymers can be recycled, most end up in landfills or oceans where pollution can significantly damage the local ecosystem.

The synthetic plastics used in food packaging are environmentally unfriendly and we have begun to explore biodegradable packaging materials. Therefore, the food industry is searching for the possibility of using biodegradable materials derived from natural sources such as cellulose and its derivatives, chitosan, starch, alginate, pectin, pullulan, gelatin, whey, soybean proteins, etc. Bacterial cellulose and other plant-derived celluloses have been used as food packaging materials, but they do not have any functional or mechanical properties. Alginate exhibits good tensile strength, good film-forming abilities, and flexibility, but it has the major drawback of a high water vapor transmission rate [3]. Gelatin is also well known for its film-forming ability but is limited to mechanical properties and thermal stability [4]. Among these biopolymers, chitosan and its derivatives have excellent film-forming properties, nontoxicity, biodegradability, antibacterial, antifungal, and metal-chelating characteristics [5]. Thus, the demand for chitin and chitosan has increased [6,7].

**Scheme 1:**
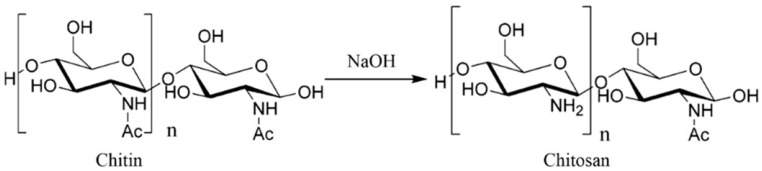
Chitosan preparation from chitin by deacetylation [8].

Chitosan is a polysaccharide found in crustacean shells that can be manufactured via the deacetylation of chitin (Scheme 1) [8]. As an alternative material, chitosan shows potential applications in many industries, such as food, agriculture, and medicine. Chitosan films and coatings have been extensively studied because they are non-toxic, antimicrobial, biodegradable, and produced from a renewable source [9,10]. The film is generally obtained using the casting method, where chitosan dissolves in a suitable solvent and simultaneously incorporates the plasticizer, the active compound, and the nanofiller of interest. Chitosan possesses antibacterial properties due to positively charged amino groups (NH_3_^+^) at the C-2 positions in the glucose monomers.

Recently, several approaches have been adopted to modify chitosan to achieve the desired food-packaging qualities. Incorporating different additives such as essential oils and plant extracts with chitosan significantly enhances the antibacterial and antioxidant properties, the current trend in a coating that improves the shelf life [11,12,13,14]. Additional modifications with starch, alginate, and pectin improve mechanical properties [15]. Furthermore, researchers improve the mechanical and physiochemical properties by incorporating bioactive additives such as polyphenols, oils, and plasticizers [16,17]. Comprising nanoscale reinforcements is another way to increase the food packaging quality [18,19].

Many attempts have been made to produce chitosan composites with nanoparticles, phenols, plant extract, metal ions and tannic acid, citric acid, etc. to enhance the pharmacological properties of the products [20,21]. However, continuous progress in this area needs more compilation. The present review focuses on different types of chitosan composites that enhance physicochemical properties and increase food quality as a packaging. This article aims to summarize the latest developments of chitosan modification as a modern food packaging.

### Antimicrobial Mechanism of Chitosan

Chitosan shows antimicrobial activity against various microorganisms, including bacteria, fungi, and algae [22]. The antimicrobial activity of chitosan includes the degree of polymerization, the host, the chitosan composition, and the substrate composition [22,23]. However, the antimicrobial activity of chitosan is still unknown and several mechanisms have been hypothesized [22]. Research shows that chitosan exhibits higher bacteriostasis than fungistasis at low temperatures (−20 °C) [24]. A hypothesized antimicrobial mechanism is that chitosan-based films form a cellophane-like structure to build a protective layer on the food surface to block external microbes [25]. The protective layer of chitosan-based films may also restrict oxygen permeability to inhibit the growth of bacteria within the food [26]. Another antimicrobial mechanism includes the adsorption of the chitosan polymers on the surface of the microbes to cause a blockage.

Chitosan contains positively charged NH_3_^+^ groups that can interact with the negatively charged phosphoryl groups leading to intercellular electrolyte leakage and killing the bacteria [27,28,29]. Chitosan can also inhibit microbes from replicating by penetrating the nuclei, binding to the DNA, and preventing the synthesis of RNA [30]. Finally, chitosan can chelate essential nutrients and metals, rendering them unavailable for microbes [20].

## 2. Chitosan Composites

Chitosan obtained from the deacetylation of chitin is a waste product of the seafood industry. The film-forming ability allows chitosan to form an edible coating or films that can improve the preservation of food products. Interactions between the cationic chitosan film microorganisms’ negatively charged membrane prevent bacteria and fungi’s growth. Pristine chitosan films have been studied for years as a potential food packaging material. However, recent research on chitosan includes chitosan-based composite films, which contain different functional components to improve the properties of films.

### 2.1. Chitosan/Essential Oil Composites

Natural essential oils extracted from plants have potential applications as environmentally friendly food preservatives in edible films. Adding essential oil to polymers increases the films’ antibacterial and antifungal activity [26,31]. Moreover, the essential oil in chitosan-based films regulates the release rate of antimicrobial agents to maintain the concentration of active compounds over an extended time. Essential oils include between 85 and 99% volatile substances, such as terpenes, terpenoids, and other alpha-aromatic and aromatic substances, and between 1 and 15% non-volatile substances; these substances are frequently utilized in the food sector [32].

On that basis, ginger essential oil (GEO) obtained from ginger enhanced the physiochemical properties of chitosan and fish sarcoplasmic protein (FSP) film. All the GEO/FSP/chitosan composite films exhibited excellent UV-barrier properties from 200 to 280 nm, decreased tensile strength, and higher elongation at break compared to pristine chitosan film. The composite film prolonged the shelf life of fillets by two days and enhanced the antimicrobial activity of chitosan films. Additionally, GEO was slowly released onto the surface of the fish to improve preservation [33]. Another chitosan-based composite film that contained essential oil of fennel (EOF), potato peel extract (PPE), and polyvinyl alcohol (PVA) improved the antioxidant and antimicrobial properties of the film. The nano chitosan-PPE-PVA-EOF composite film enhanced mechanical properties and extended the shelf life of food. The composite film degraded entirely within two days by *Pseudomonas putida* while the chitosan control film took about one week [34]. Furthermore, composite films based on chitosan and honeysuckle flower extract (HFE) enhanced antioxidant activity, antimicrobial activity, and reduced water vapor permeability (WVP). With the addition of HFE (30%), the DPPH scavenging activity and *E. coli* inhibition zone increased. However, mechanical tests showed that incorporating HFE reduced the tensile strength and elongation at break. The composite films have great potential for antimicrobial packaging for highly perishable foods [35].

**Figure 1:**
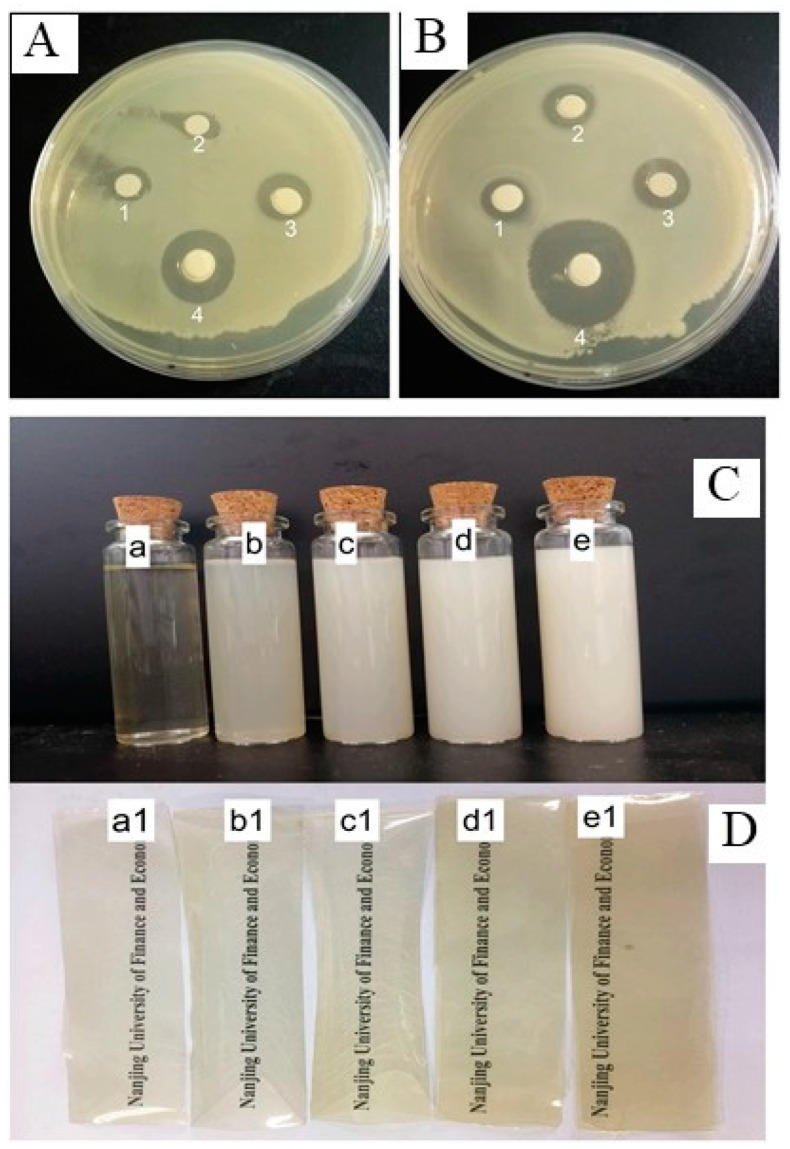
Antimicrobial activity of CEO emulsions against *E. coli* (**A**) and *S. aureus* (**B**). GA/CEO, GA/CEO-U, OSA-GA/CEO, OSA-GA/CEO-U represents 1–4, respectively. Visual appearance of film-forming dispersions (**C**) and films (**D**) with different ratios of CEO emulsions: a, a1—0% emulsion; b, b1—5% emulsion; c, c1—10% emulsion; d, d1—15% emulsion; e, e1—20% emulsion [36].

The essential oil extracted from cinnamon has been getting attention due to its antimicrobial properties. Octenyl succinic anhydride (OSA) and gum arabic (GA) stabilized with cinnamon essential (CEO) can be incorporated into chitosan-based films to increase the emulsion capacity of OSA and GA. CEO emulsions into the chitosan films improved antimicrobial activity against *E. coli* and *S. aureus*. However, it reduced the tensile strength of the film. The OSA-GA stabilized CEO emulsions into CS films could be used to protect edible films and increase durability (Figure 1) [36]. Similarly, chitosan film containing thyme essential oil enhanced the film’s mechanical, thermal, and antibacterial properties. Moreover, combing of calcium chloride, zinc oxide (ZnO), poly ethylene glycol (PEG), and nano clay (NC) in chitosan-based films preserved the quality of sweet cherries by conserving higher titratable acidity, total soluble solids, and reduced weight loss [37].

### 2.2. Polyphenolic/Chitosan Composite

Chitosan film without composite is hydrophilic and has weaker mechanical, thermal, and water vapor barrier characteristics than synthetic composites. To increase the use of chitosan films, nature extracts, which often contain a variety of functional activities, can be added to the film as active components and fillers. Several phenolic compounds (ferulic acid, gallic acid, tannic acid, quercetin, and curcumin) have recently been reported as chitosan-based composite films [38,39,40,41,42,43]. These phenolic compounds could enhance various properties, including the mechanical strength of chitosan films [39,43].

**Figure 2:**
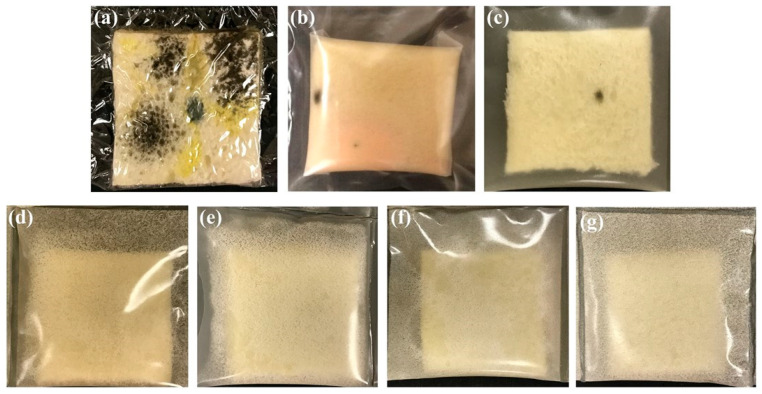
Bread samples packaged by (**a**) PE, (**b**) PCL/chitosan, (**c**) GFSE content 0.5, (**d**) 1.0, (**e**) 1.5, (**f**) 2.0, and (**g**) 2.5 films at 24 °C, for one week [47].

The phenolic compound (gallic acid, procyanidins, catechin, and epicatechin) from grape seed extract (GSE) can have antioxidant and antimicrobial activity. GSE and carvacrol incorporated in chitosan films extended the shelf life of refrigerated salmon up to one week [44]. A mathematical model study also proved that gallic acid release from chitosan/carvacrol films improves food preservation and shelf-life extension of water-based food products at different temperatures [45]. Similarly, in the study of Sogut et al., the GSE/chitosan films exhibited higher elastic modulus and elongation at break compared to chitosan films. The antioxidant activity, light barrier properties, and antibacterial activity also increased as the GSE content increased in the chitosan film. Chitosan films incorporated with GSE exhibited superior antimicrobial activity against *Listeria monocytogenes*, *Escherichia coli*, and *Staphylococcus aureus.* Moreover, composite chitosan films with 15% GSE inhibited the total mesophilic aerobic bacteria and coliform foods [46]. The GSE with poly(ε-caprolactone) (PCL) and chitosan was reported as an antimicrobial packaging for food by inhibiting the growth of *E. coli* and mold, respectively. The pit morphology of chitosan films increased as the concentration of GFSE increased due to the incorporation of glycerol in the GFSE. These pits facilitate the release of GFSE out of the composite films and can suppress microbial growth. The GFSE chitosan composite films still exhibited adequate tensile strength and flexibility but decreased the film’s resistance to breakage and elasticity. Furthermore, no mold growth was observed on the bread packaged with films containing GFSE (Figure 2) [47].

Another polyphenol group from kombucha tea (KT) embedded with chitosan films improved antimicrobial and antioxidant activities, extending the shelf life and lowering the minced beef’s lipid oxidation by three days. KT significantly enhanced the antioxidant activity of chitosan-based films. Food packaging material should ideally exhibit low light transmittance. UV–vis spectroscopy analysis between 300 nm and 800 nm determined KT significantly decreased UV and visible light’s transmittance. Adding KT also considerably increased the water solubility and reduced the water vapor permeability of chitosan films [1]. Karača et al. fabricated an edible film that contained coca (*Theobroma cacao* L.) powder using chitosan, alginate, and pectin with whey protein. The resultant plain alginate films exhibited the highest elongation at break. Adding proteins caused the release of polyphenols to be prolonged and enhanced the functional properties. The plain alginate and pectin films exhibited the highest total phenol content (TPC) compared to chitosan-based films, alginate- and pectin-based films. Further, the sensory score was reported to be highest in alginate-based edible films based on acceptability, appearance, color, transparency, and elasticity [48].

Protocatechuic acid (PA), a natural phenolic antioxidant grafted with chitosan (PA-g-CS), exhibited higher water solubility and lower moisture content than chitosan films. The film exhibited greater tensile strength and elongation at break compared to chitosan films. Furthermore, the PA-g-CS films showed lower transmittance of light around 300 nm, and the decomposition of the film was lower between 30 °C and 167 °C. Finally, PA-G-CS also exhibited higher antioxidant activity than chitosan films. The free radical scavenging activity increased as the PA content increased [49]. The syringic acid/chitosan composite film effectively preserved quail egg coatings by inhibiting bacterial growth. The obtained film exhibited increased density, water solubility, and opacity, and decreased water vapor permeability and water content [50].

### 2.3. Polysaccharide/Chitosan Composite

Reports have shown that polysaccharides can be blended with chitosan to develop chitosan-based functional films. The advantages of using starch include low cost, availability, biodegradability, and renewability. Additionally, starch and chitosan possess good film-forming capacities, which enable the formation of a composite film [51]. The obtained film enhanced antioxidant activity and water vapor barrier property, while lowering bacterial adhesion on the packaging. These properties show that the composite has potential applications as an active packing film for foods [52,53].

Bhardwaj et al. investigated the properties of food-grade cellulose-based papers made from sugarcane bagasse and corn husk coated with chitosan and beeswax–chitosan emulsion. The WC (beeswax–chitosan emulsion coated) and CC (chitosan coated) paper reduced yeast and mold growth by 8 to 12 days, depending on the temperature. In addition, the enhanced barrier properties against air and moisture are attributed to better bread quality [54]. The chitosan/cellulose nanocrystals (CNC) exhibited antimicrobial activity against the gram-negative, gram-positive bacterial, and fungicidal activity. The chitosan films also reduced Pseudomonas and Enterobacteriaceae bacteria growth in meat. The obtained chitosan films exhibited enhanced oxygen barrier and thermal stability properties; however, the water permeability stayed the same. Additionally, chitosan/CNC films exhibited increased tensile strength and Young’s modulus [55]. In another study, CNC added to chitosan solution was observed to have improved the water resistance of the films prepared by the solvent casting method. The nanochitosan (NCH)-CNC suspension was cast into a petri-dish and dried at 50% relative humidity for two days. The resultant NCH-CNC films exhibited a smooth and continuous surface. The film morphology was reported to be smoother in nanoform and the film thickness was decreased by the incorporating of nanocellulose [56].

In the continuous growth of biodegradable materials, starch shows the potential to develop active food packaging material due to its low cost, availability, functionality, and, most importantly, biodegradability. Starch-based films are often odorless, tasteless, colorless, nontoxic, and biodegradable. However, compared to synthetic polymers, starch films have poor mechanical and barrier qualities, restricting their use in food packaging. Therefore, scientists have been investigating retaining starch characteristics by blending with the other natural biopolymers. The intermolecular bonds between the amino functional groups of chitosan improved many food packaging properties such as tensile strength, thermal stability, hydrophobicity, water adsorption, and gas barrier properties [57]. Corn starch and chitosan film prepared via the casting method exhibited excellent elongation at break and tensile strength [58]. Thermoplastic corn starch (TPS) and chitosan oligomer (CO) composite films extended food shelf life. The chitosan oligomers diffused through the polymer towards the food surface acting as an antimicrobial agent. Results showed that the film could preserve perishable foods for one week. The resultant TPS/CO composite film exhibited good antimicrobial capability and suggested an efficient way to inhibit microbial development [59].

**Figure 3:**
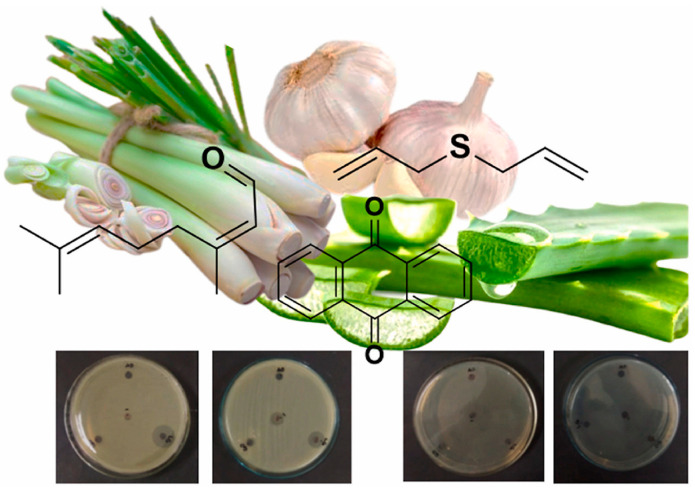
Schematic representation of chitosan–starch based composite film with antibacterial activity [61].

Sugar palm starch (SPS) with chitosan and extra virgin olive oil (EVOO) composite film exhibited the lowest second-stage degradation temperature (184.2 °C). In comparison, chitosan/SPS-EVOO (2%) showed the highest second-stage degradation temperature (223.1 °C) and had the most optimal improvement in elongation at break and tensile strength [60]. The chitosan–*Dioscorea alata* starch composite film developed by response surface methodology (RSM) achieved desirable food packaging properties such as moisture content, biodegradability, elastic modulus, and tensile strength. Further, adding essential oil from garlic, aloevera, and lemon grass increased the antibacterial properties against *E. coli*, *Salmonella typhi*, *S. epidermidis*, and *S. aureus* (Figure 3) [61]. Furthermore, the composite film of starch–chitosan and montmorillonite filler nanoparticles significantly increased tensile strength and improved water vapor barrier properties [62].

Incorporating chitosan nanoparticles (CNP) into starch films acted as a natural filler and improved the film’s mechanical properties and water sorption [63]. The starch/CNP nanocomposite films inhibited the growth of *S. aureus*, *B. cereus*, *E. coli*, and *S. typhimurium* and extended the shelf life of perishable foods [64]. Similarly, Zhao et al. added chitosan and gallic acid into cassava starch via subcritical water technology to produce a composite film. Hydrogen bonds and crystallinity affected the mechanical properties of each respective film. Results showed that chitosan–starch films exhibited the highest tensile strength and elongation (100.1%). Additionally, cassava starch was found to be a capable carrier of antioxidants and antimicrobials which can extend the shelf life of foods. The chitosan/gallic acid/cassava starch composite film elongated the shelf life of cooked ham for 25 days and may have a potential application as a food packaging material [65].

The other polysaccharides, such as pectin, can also be blended with chitosan to prepare a composite film that is stable and uniform due to the intermolecular interactions between the oppositely charged ions. A film that combined tea polyphenols (TP), pectin, and chitosan (CPFT) increased the water vapor permeability and moisture content. Adding TP improved antioxidant activity and reduced bacterial growth [66]. Similarly, Schnell et al. prepared a film that contained colloidal suspensions of cationic polyelectrolyte complex (CatPEC) of xylan (Xyl) and chitosan. The obtained film exhibited lower water vapor transmission rates and oxygen permeability. Moreover, it inhibited *E. coli* and *S. aureus* growth [67].

**Figure 4:**
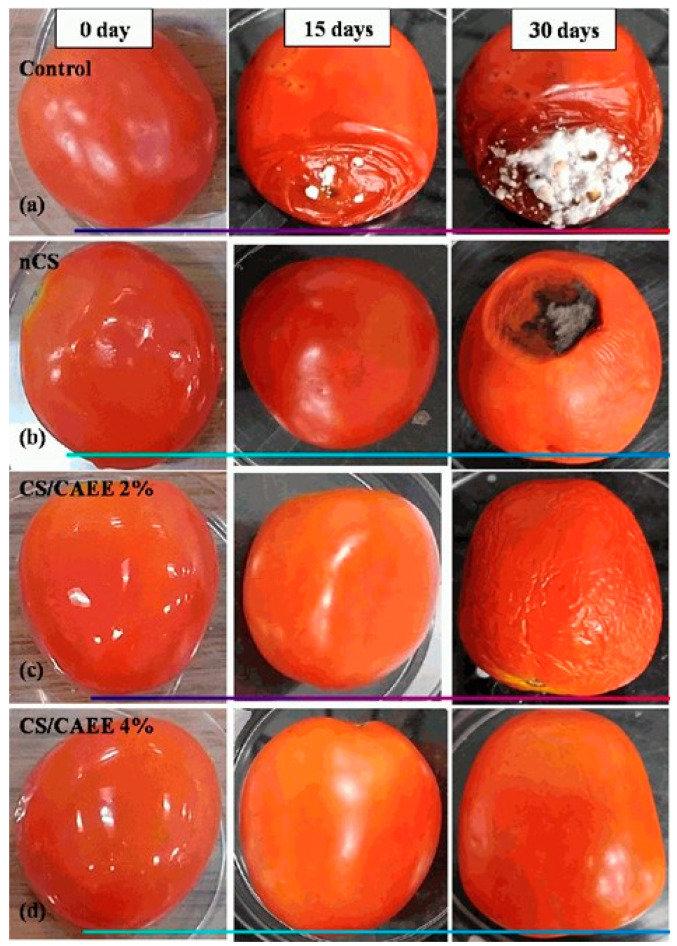
Photograph of tomato: (**a**) control, (**b**) nCS, (**c**) CS/CAEE 2%, (**d**) CS/CAEE 4%, at a specific storage day [70].

Konjac glucomannan (KGM) is a polysaccharide and water-soluble dietary fiber, derived from *Amorphophallus konjac*. KGM combined with TEMPO-ChNCS (TEMPO-oxidized chitin nanocrystal) increased tensile strength and reduced water vapor permeability [68]. Similarly, the composite of epigallocatechin gallate (EGCG) with KGM/carboxymethyl chitosan (KGM/CMCS) demonstrated desirable characteristics of superior antioxidant properties and antibacterial properties that are essential for food packaging. EGCG enhanced the water vapor barrier properties, increased thermal stability, increased tensile strength, and increased UV-light barrier properties [69]. Further, a de-oiled crude green algal ethanolic extract (CAEE) was incorporated into chitosan films to minimize postharvest loss and prolong the shelf life of tomatoes (Figure 4) [70]. Chitosan composite with polysaccharides improves their physical and mechanical properties, depicted in Table 1.

### 2.4. Gelatin/Chitosan Composite

Gelatin is a protein obtained from animal collagen that blends with chitosan used to make composite films that exhibit improved mechanical properties, water barrier properties, and light barrier properties [71,72,73,74].

Perishable foods treated with chitosan–gelatin edible films exhibited low microbial decay and longer shelf life [75]. The shelf-life of a carrot piece can be increased upto ten days, using a composite film of chitosan and gelatin [76]. Furthermore, the gelatin/chitosan composite with phenolic antioxidants (gallic and trans-cinnamic acids) exhibited significantly higher reducing power (RP) and iron chelating power (ICP) compared to the trans-cinnamic acid/gelatin/chitosan films. The kinetic release of phenolic compound strongly influenced the antioxidant activity, RP, ICP, and antimicrobial activity of the films [77].

**Figure 5:**
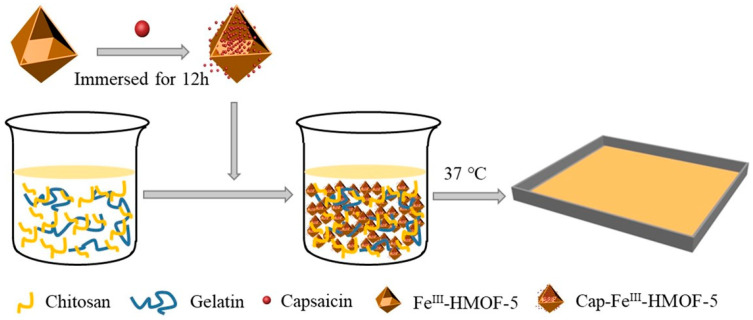
Schematic representation of Cap-Fe^III^-HMOF-5 modified gel/chi film preparation [81].

A composite film of cinnamon essential oil (CEO), chitosan, and gelatin via the casting method exhibited UV-light barrier properties. The composite film also showed greater elongation at break but a decreased tensile strength compared to CS films. Additionally, the results found that gelatin and CEO enhanced thermal stability while reducing wettability and crystallinity. The composite film also exhibited excellent antibacterial activity against *E. coli* and *S. aureus* [78]. Halim et al. showed that incorporating tannic acid into CS, gelatin, and methylcellulose (MC) increased the antibacterial properties of the composite film against *E. coli* and *S. aureus.* MC exhibited the lowest weight loss % while chitosan exhibited the highest. Additionally, the incorporation of tannic acid significantly reduced light transmittance which is a better preservative [79]. Combining nano-cellulose into chitosan, gelatin, and starch improved the anti-fungal, mechanical, and water barrier properties. With the chitosan amount, food preservation, Young’s modulus, and tensile strength increased while the elongation at break decreased. However, Young’s modulus and tensile strength decreased as the nanocellulose content increased. Additionally, composite films that contained starch nanocellulose were more brittle due to the rigid behavior of NCC compared to gelatin. Additionally, the nanocomposite film containing ~5% NCC content of starch-based film exhibited high transmittance of UV light [80]. Furthermore, the antibacterial performance of gelatin/chitosan film was enhanced by incorporating capsaicin (Cap) via FeIII doped hollow metal–organic framework (Fe^III^-HMOF-5) nanocarriers. Incorporating Cap- Fe^III^-HMOF-5 increased tensile strength and reduced elongation at break. Moreover, due to the hydrophilic properties of Cap- Fe^III^-HMOF-5, water vapor permeability decreased as the content increased (Figure 5) [81]. The gelatin/chitosan film improves the physical and mechanical properties, depicted in Table 2.

### 2.5. Polyvinyl Alcohol/Chitosan Composite

Food packages containing chitosan kept the food fresh without a freezer for some time while degrading and preventing future soil pollution. However, the fast dissolution in acidic solution and lack of flexibility of chitosan films are its drawbacks. Therefore, creating an affordable, biodegradable polymer film for food packaging is essential. Polyvinyl alcohol (PVA) has numerous advantages for food packaging, including high hydrophilicity, outstanding chemical stability, and exceptional film-forming abilities. Therefore, the current research on the composite of PVA with chitosan is gaining attention.

**Figure 6:**
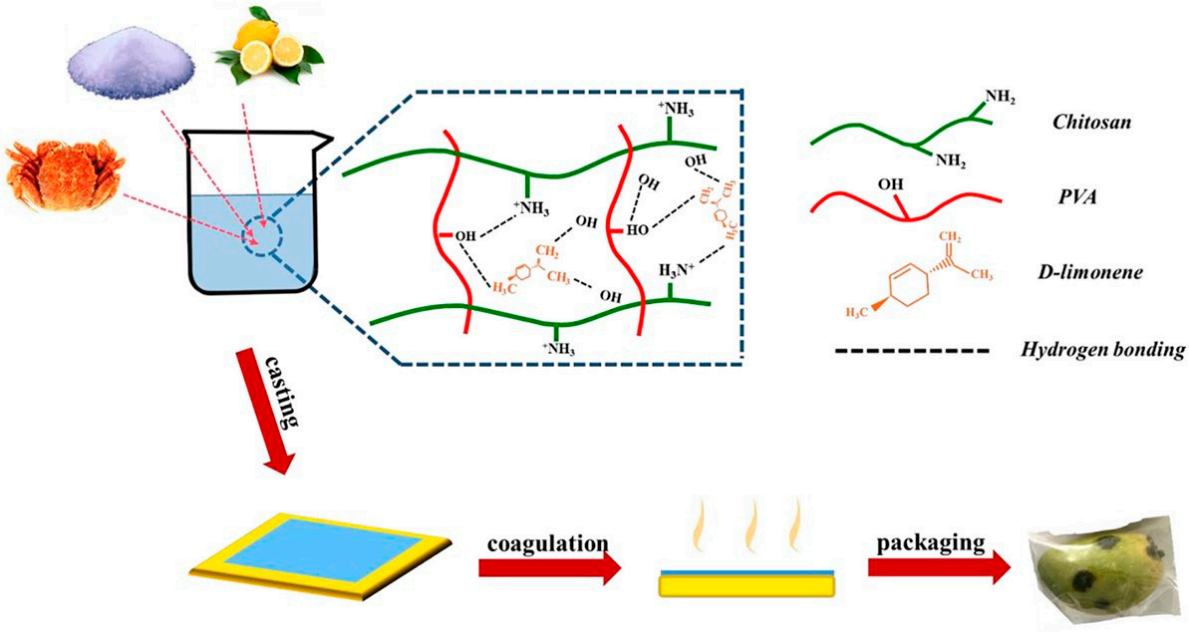
Schematic diagram for the preparation of PVA/CS/DL composite films that increased the shelf life of mangoes [85].

Hydrogen bonding can cause entanglements between PVA and quaternary ammonium chitosan (QAC), forming a composite film with enhanced mechanical properties [83]. A study by Hu et al. showed that the QAC/PVA composite film significantly increased water vapor permeability, tensile strength, and elongation at break. The films reduced peroxide levels and thiobarbituric acid levels in peanut oil and stored it for 28 days at 37 °C [84]. Similarly, the composite of PVA, chitosan, and various levels of D-Limonene (DL) film exhibited the most optimal tensile strength and water vapor permeability. The composite films also inhibited *E. coli* and *S. aureus* growth and increased the shelf life of mangoes. The film significantly slowed the decay process (Figure 6) [85].

A chitosan/polyvinylpyrrolidone (PVP) composite film enriched with nanoparticles (NP) from plant extracts could detect fungi infection progress in strawberries. This phytopathogenic fungus was detected faster using NP added to a plastic package containing inoculated strawberries as it changed their absorbance and transmittance during *R. stolonifer* growth [86]. A biodegradable film made of PVA/chitosan can be improved with silica as a safety candidate. By hydrogen bonding between silica and PVA or chitosan, Yu et al. improved tensile strength of biodegradable PVA/chitosan films by as much as 45%. The preservation time was also extended thrice by reducing oxygen permeability and moisture by 25.58% and 10.2%, respectively [87].

Similarly, chitosan/PVA-based films with sulfosuccinic acid (SSA) fabricated by casting method and UV curing process exhibited significantly higher tensile strength while reduced elongation at break. The obtained film improved mechanical, thermal, and water barrier properties by adding glycerol and xylitol to 20–180%. Apple coated with the film reduced the decomposition time by up to 70 days compared to uncoated apples. The biodegradability of the film was found to have degraded by about 40–65% after 220 days [88]. Furthermore, sodium lactate loaded in chitosan–PVA/montmorillonite (NaL-CS/PVA/MMT) showed increased water sorption activity with MMT content. However, the incorporation of MMT decreased water vapor permeability, and significantly reduced oxygen and carbon dioxide permeability. Additionally, it inhibited the growth of *E. coli* and well-controlled NaL release, which was dependent on the pH value of the aqueous solution and ionic strength. The composite film exhibited barrier properties suitable for food packaging [89].

### 2.6. Metal/Metal Oxide Nanoparticles/Chitosan Composite

Several attempts have been undertaken to modify the characteristics of chitosan for packaging materials by mixing them with plasticizers or nanoparticle fillers. Compared to the original polymers or traditional composites, the nanocomposites were reported to have superior barrier, mechanical properties, and heat-resistant capabilities, and suggest potential applications for food packaging.

Silver nanoparticles (AgNP) exhibit antimicrobial properties against various microorganisms and can combine with chitosan for food packaging. Thus, AgNP was used to form catechol-modified chitosan AgNP (CSCT AgNPs) and gelatin (CSCT/G/AgNP) composite films. Results showed gelatin improved the mechanical and water barrier properties while AgNP enhanced antibacterial properties against *S. aureus* and *E. coli.* The bactericidal ratio increased significantly as the AgNP content increased. The improved mechanical properties, physiochemical, and antibacterial properties indicated potential application as active food packaging material [82]. In another study, chitosan-based films modified with an azopolymer and AgNP also have the potential in food packaging applications. Further it showed that composite film also exhibited lower hydrophilicity and more excellent thermal stability than pristine chitosan films. Mechanical testing showed that the tensile strength and elastic modulus increased for blend and nanocomposite films. The chitosan-based modified azopolymer with AgNP exhibited enhanced light barrier properties, thermal stability, and mechanical properties [90].

**Figure 7:**
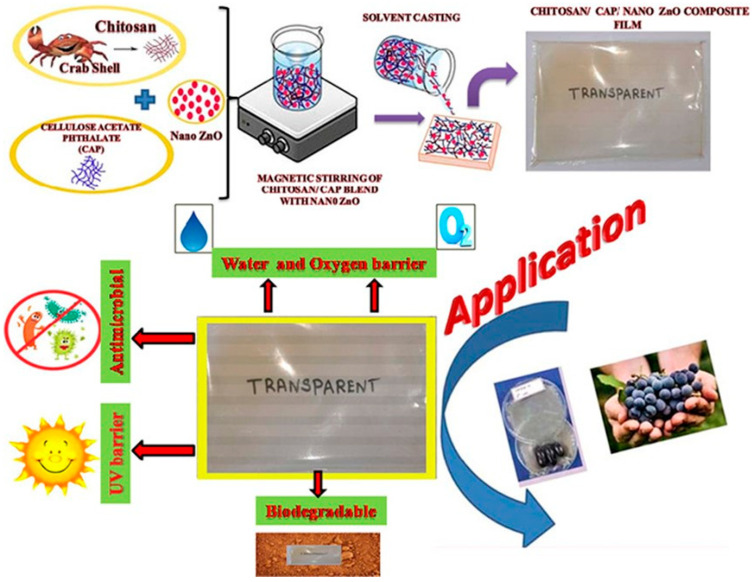
Schematic diagram of CS/CAP and ZnO nanoparticle film preparation and applications [91].

Zinc oxide (ZnO) can also be added to chitosan films to enhance physiochemical and biological properties. ZnONP embedded into chitosan cellulose acetate phthalate (CAP) films improved mechanical, barrier, and antimicrobial properties. The chitosan–CAP films with 5% *w*/*w* nanoZnO (CCZ) were reported to have the most optimal strength and stiffness, which significantly (9 days) extended the shelf life of black grapefruits. Additionally, the oxygen and water vapor transmission rates of CCZ films were substantially lower with larger inhibition zones (Figure 7) [91]. ZnONP/chitosan composite films exhibited greater thermal stability at high temperatures due to the hydrogen bonds between the ZnO and chitosan [92]. A study by Souze et al. showed the ZnONP synthesized by using apple peel extracts incorporated into chitosan films also improved the antimicrobial activity of composite films. Experiments with fresh poultry showed the shelf life was extended due to the film decreasing the deterioration rate [93].

Adding melissa essential oil (MEO) into the ZnO/chitosan composite films enhanced mechanical properties. MEO enhanced antibacterial activity while ZnO enhanced the crystalline structure of chitosan films. As the composition of ZnO and MEO increased, the transparency of the film also increased [94]. It was also reported that when chitosan and ZnO nanoparticles were incorporated into gallic acid films (Ch-ZnO@gal) it improved the mechanical, physiochemical, and antibacterial properties. Resultant Ch-ZnO@gal composite films were reported to exhibit significantly greater tensile strength than chitosan films. However, the elongation at break of Ch-ZnO@gal was lower than the chitosan control group. The incorporation of ZnO@gal enhanced the light barrier properties, water vapor permeability, and oxygen permeability. Further antioxidant activity showed that the antioxidant activity increased as the concentration of ZnO@gal increased. Finally, with the concentration of ZnO@gal, the antibacterial property increased. The results seem to suggest that ZnO@gal has potential applications to improve biocomposite chitosan films for food packaging applications [95]. Wang et al. studied a composite film of carboxymethyl chitosan (CMCS), carboxymethyl cellulose sodium (CMC) polylactic acid (PLA), and ZnO NP. Multilayers of the composite film showed that the oxygen barrier properties were significantly (99%) greater than a layer of PLA coating. Additional results also showed that films with ZnO nanoparticles exhibited water, oxygen, heptane vapor, oil barrier, and high antibacterial properties [96].

Sulfur nanoparticles (SNP) are known to be great as antimicrobial agents. Shankar et al. developed a chitosan/SNP composite with greater tensile strength and elastic modulus. The tensile strength of the chitosan/SNP films increased with the SNP content. Further mechanical tests showed that SNP decreased the elongation at break of the chitosan films. Thermogravimetric analysis showed that chitosan/SNP composite films exhibited greater thermal stability than chitosan films. The results found that chitosan/SNP composite films completely inhibited the growth of *E. coli* and *L. monocytogenes* within 6 h and 12 h, which may have potential applications for active food-packaging material [97]. Another study prepared chitosan and PVA coatings with ferulic acid as a cross-linking agent using layer-by-layer assembly technology. Due to the homogenous spread of nanoparticles, the tensile strength, water resistance, antioxidant, and antibacterial activities improved. Experiments showed that the resultant films could extend the shelf life of cherry tomatoes [98].

### 2.7. Clay/Chitosan Composite

Montmorillonite (MMT), a potential adsorbent in food packaging films, was considered as a nanocarrier of essential oil [99]. Giannakas et al. adsorbed essential oil into sodium montmorillonite (NaMt) or organomodified-montmorillonite (OrgMt) and prepared a composite film with chitosan. The nanocomposite films exhibited lower water adsorption values, the highest barrier properties, and great antimicrobial activity against *E. coli* The nanocomposite films prepared from OrgMt via the modified clay exhibited better antioxidant activity than films prepared with NaMt. The Cs/OrgMt/TO nanocomposite films were effective food packaging films that extended the shelf life of foods [100].

Souza et al. investigated the physical and morphological properties of chitosan/MMT (CS/MMT) nanocomposite films activated with ginger essential oil (GEO). The chitosan composite films began to degrade significantly at very high temperatures between 283.5 °C and 290.8 °C due to degradation of chitosan. Experimental results found that incorporating of GEO and MMT enhanced the barrier properties against light, gases, and water vapor [101]. Moreover, chitosan/Na-MMT increased the shelf life of cherry tomatoes. The film significantly reduced the mass loss and nutrient loss of cherry tomatoes. As a result of the incorporation of MMT into the composite films, the films showed improved swelling resistance and elongation at break (68%). In contrast, the films without MMT exhibited poorer structural and thermal properties [102]. CMC-MMT incorporated with TiO_2_ showed antibacterial inhibition for both gram-negative and gram-positive bacteria. Also, the tensile strength of the film containing TiO_2_ nanoparticles increased [103].

The hydrogen bond between chitosan and the Si-O bonds of nanoclay influenced changes in the mechanical properties of the materials. The nanocomposite films with chitosan/tween 80/rosehip seed oil (RSO) formed by dispersed MMT nano clay C30B via casting film-forming emulsions exhibited significantly higher elasticity. The composite film showed suitable CO_2_ barrier properties for food packaging material. The composite films also demonstrated antimicrobial activity against *E. coli*, *Salmonella typhymurium*, and *Bacillus cereus* that increased the shelf life of perishable foods. The RSO improved the mechanical, gas, and water vapor barrier properties and antioxidant activity [104]. Additional studies also showed that MMT and tocopherol improved chitosan film’s mechanical properties, thermal stability, and antioxidant activity [105].

**Figure 8:**
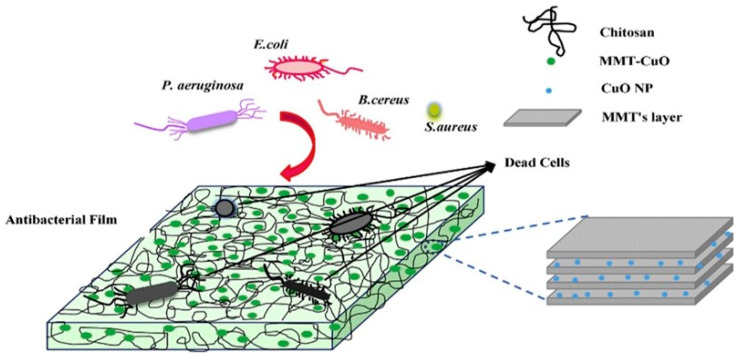
Graphical representation showing antimicrobial properties of MMT-CuO nanocomposites [107].

Colorimetric oxygen indicator film made of chitosan/MMT grafted with cyclodextrin (CD) with methylene blue/glucose system is used for packaging foods. When oxygen is present, the MB–glucose redox system causes the film to change color. MMT and CD grafting onto chitosan increased hydrophilicity and enhanced the film color change. The films were promising for food packaging because of their high color sensitivity to high relative humidity. PET films may also delay the color change to blue with a low oxygen permeability. In addition, the film can elongate the storage time for three weeks in a refrigerator [106]. When MMT–copper oxide nanocomposites (MMT-CuO) were incorporated into the chitosan matrix, it improved the optical, mechanical, and antibacterial properties. In contrast, the water solubility, UV transition, water vapor, and oxygen permeability decreased. The MMT-CuO nanocomposites had little effect on the transparency of the films and increased the tensile strength and percent elongation. As a result, chitosan-MMT-CuO nanocomposite films have potential applications in active food packaging (Figure 8) [107].

A study by Yan et al. showed that the α-tocopherol-chitosan nanoparticles/chitosan/montmorillonite (TOC-CSNPs/CS/MMT) composite films can be used for food packaging applications with improved physiochemical properties and antioxidant activity. The water solubility of the TOC-CSNPs/CS/MMT composite films decreased with the TOC-CSNP content. Films with 10% TOC-CSNP exhibited the lowest water solubility. Furthermore, the swelling ratio decreased as the TOC-CSNP content increased. Antioxidant evaluation of the composite films found that the composite films exhibited significantly higher radical scavenging activity than CS/MMT and CS films [108].

### 2.8. Carbon/Chitosan Composite

Recently, graphene has been utilized in the food packaging industry due to its potent antimicrobial properties. Graphene oxide (GO) has the unique property of being homogenously dispersed into chitosan polymer nanocomposite films. The GO–chitosan nanocomposite films significantly improved the thermal stability and mechanical properties. Crosslinking between chitosan and GO may have significant effects on mechanical properties. The incorporation of GO increased the tensile strength and thermal properties of the chitosan film. Moreover, the antimicrobial properties against *E. coli* and *B. subtillis* were also contributed to by the incorporation of GO [109]. A laser-induced micropore-based modified atmosphere packaging and carbon-dot/chitosan coat exhibited good preservation of food flavor components such as alcohol, ketones, and aldehydes [110].

## 3. Conclusions and Future Prospects

The awareness of environmental problems caused by plastic packaging has increased interest in environmentally friendly packaging materials. The seafood industry generates large amounts of crustacean shell waste every year. In addition to chitin, this waste produces chitosan using various methods. Chitosan is substantially less expensive than other biopolymers since it is derived from a bio-waste product utilizing various energy-efficient techniques. Chitosan, one of the polysaccharides, has undergone significant research and has been used to create biodegradable food packaging. Due to its antibacterial properties, chitosan is frequently utilized as antimicrobial films, edible protective coatings, dipping, and spraying for food items. Antimicrobial packaging is crucial, and can kill or inhibit food-contaminating pathogenic bacteria. Researchers have combined chitosan with other biopolymers and nanocomposites to improve mechanical properties, optical properties, barrier properties, and thermal stability. We have selected a few of the best composites with improved essential properties for food packaging in Table 3. However, more research is required to get this biopolymer to industrial levels for food packaging applications. A few aspects should be considered for future developments as follows: (i) degradation of the films in the real environment; (ii) the toxicity of chitosan-based film; and (iii) active and intelligent packaging system. Over the past ten years, much work has gone into creating and testing antimicrobial films to increase food safety and shelf life. This review briefly overviews recent developments in creating chitosan composite films for food packaging.

## Figures and Tables

**Table 1:** Starch incorporated chitosan film with different additives.

Composite	Functional Properties		Physical Properties		References
	Antimicrobial Capacity	Antioxidant Activity	Thickness	Mechanical	
Chitosan/corn starch/glycerol	----	----	----	Tensile strength increased up to 2.24 times compared to pure chitosan	[58]
Chitosan/cassava starch/gallic acid/glycerol	Delayed microbial growth for two weeks	No significant changes compared to Trolox equivalent performance	----	Tensile strength increased from 0.51 to 0.83 MPa with the chitosan content	[65]
Chitosan/starch/sodium tripolyphosphate	----	----	20–100 nm diameter	----	[63]
Chitosan/sugar palm starch/extra virgin olive oil (EVOO)	Inhibitory activity against *E. coli* and *S.aureus*	Increased significantly on the addition of EVOO up to 2.0%	Varied based on EVOO content	Tensile strength increased up to 2581 MPa with the addition of 2% EVOO	[60]
Chitosan nanoparticle/tapioca starch/glycerol/sodium tripolyphosphate	Growth inhibition of *B. cereus*, *S. aureus*, *E. coli*	----	----	----	[64]

**Table 2:** Gelatin incorporated chitosan film with different additives.

Composite	Functional Properties		Physical Properties		References
	Antimicrobial Capacity	Antioxidant Activity	Physicochemical Properties	Mechanical Properties	
Chitosan/gelatin/methylcellulose/tannic acid	Improved antimicrobial activity against *E. coli* and *S. aureus*	----	Improved water vapor barrier and oxygen barrier properties	Tensile strength decreased from 27.3 to 7.26 MPa with the addition of tannic acid	[79]
Nano-cellulose/gelatin/chitosan/starch matrices	----	----	Increased UV transparency	Young modulus increased up to 18,300 MPa with 10% of nanocellulose content	[80]
Chitosan/gelatin/cinnamon essential oil	Improved antibacterial activity against *E. coli* and *S. aureus*	----	Lower water vapor permeability and higher visible light transmission	Tensile strength increased up to 52.31 ± 1.40 MPa compared to pristine chitosan 43.49 ± 2.48 MPa	[78]
Chitosan/fish gelatin/gallic acid/trans-cinnamic acids	Strongly decreased bacterial growth of *E. coli*	Increased for films with gallic acid only	----	----	[77]
Gelatin/chitosan/Cap- Fe^III^-HMOF-5	Improved activity against *E. coli*	----	Enhanced water vapor permeability and UV block properties	Tensile strength increased up to 46.05 ± 0.21 MPa with the addition of MOF	[81]
Catechol modified chitosan/gelatin/silver nanoparticle	Inhibitory activity against *E. coli* and *S. aureus*	Increased	Improved water vapor resistance	Tensile strength increased up to 25.38 ± 0.98 when compared to pure chitosan 9.25 MPa	[82]

**Table 3:** A few of the best chitosan composites with improved essential properties for food packaging.

Composite	References
Chitosan/ZnO/CaCl_2_/NC/PEG/thyme oil	[37]
Chitosan/protocatechuic acid	[49]
Chitosan/cornstarch	[57]
Chitosan/sugar palm starch/olive oil	[60]
Chitosan/discorea alata starch/glycerol/essential oil	[61]
Chitosan/cassava starch/gallic acid	[65]
Carboxymethyl chitosan/Konjac glucomannan/epigallocatechin gallate	[69]
Quaternary ammonium chitosan/polyvinyl alcohol	[84]
Chitosan/azopolymer/silver nanoparticle	[90]
Chitosan/zinc oxide nanoparticles loaded gallic acid	[95]
Chitosan/polyvinyl alcohol/ferulic acid	[98]

## Data Availability

Not applicable.
